# Travel intention during the COVID-19 epidemic: The influence of institutional and interpersonal trust

**DOI:** 10.3389/fpsyg.2022.1015900

**Published:** 2022-10-12

**Authors:** Wenyong Li, Gang Chen, Lunwen Wu, Yanling Zeng, Jing Wei, Yao Liu

**Affiliations:** ^1^School of Business Administration, Faculty of Business Administration, Southwestern University of Finance and Economics, Chengdu, Sichuan, China; ^2^School of Business, Anhui University of Technology, Maanshan, Anhui, China; ^3^School of Business Administration, Sichuan Vocational College of Finance and Economics, Chengdu, China

**Keywords:** institutional trust, interpersonal trust, travel intention, health risk perception, safety self-efficacy, psychological resilience

## Abstract

The global pandemic, COVID-19, has dealt a heavy blow to the tourism industry. Therefore, exploring the mechanisms influencing travel intention in the post-epidemic era can help provide management insights for the recovery of the travel market. Relying on the logic of social cognition theory, we conducted an empirical analysis from the perspective of trust and found that institutional trust and interpersonal trust can positively predict travel intention in the context of the epidemic, while travelers’ health risk perception and safety self-efficacy mediate the relationship between trust and travel intention. Moreover, we verified the moderating role of tourists’ psychological resilience. Further, the study confirms that China’s active prevention policy not only reduces the physical health harm caused by the epidemic, but also effectively increases individuals’ institutional trust in a proactive government. Through China’s active anti-epidemic policy, individuals were able to counteract the negative impact of the COVID 19 epidemic on their travel intention. Further, theoretical and practical implications are discussed.

## Introduction

A major public health event, COVID-19 has caused a great deal of concern around the globe. According to the United Nations World Tourism Organization (UNWTO), global travel arrivals rose by 4% in 2021 over 2020, but were still 72% lower than in 2019, the year before the outbreak. Despite a slight increase in travel revenue from $1.6 trillion in 2020 to $1.9 trillion in 2021, global travel revenue remains substantially below the $3.5 trillion recorded in 2019. Several factors contribute to the slow pace of travel recovery, including the extent of travel restrictions, the level of vaccination rates, and traveler awareness (Bradbury-Jones and Isham, 2020). A strong government prevention and control program has helped to contain the spread of the epidemic in China and some other countries or regions of the world. Yet, the persistence and recurrence of the COVID-19 epidemic suggest that we will be forced to live with the epidemic for a long time and adjust to life following the epidemics (Kim and Su, 2020). When society’s public health is threatened by risk and uncertainty events, governments may develop proven measures to guide travelers’ perceptions and intentions (Nakayachi and Cvetkovich, 2010), especially when major public health events threaten society’s public health often have far-reaching effects on travelers’ consumption confidence, consumption decisions, and consumption behavior. As public health awareness has increased, individuals have changed their travel patterns as well as their life habits in response to the COVID-19 epidemic. China had long been the world’s top source of outbound travel and the fourth largest recipient of inbound travel, but the epidemic brought inbound and outbound travel to an abrupt halt. It has been a challenging policy undertaking for the Chinese government since 2020 to find a way to balance the health of its 1.4 billion citizens with the economic recovery of domestic travel. Travelers in the post-epidemic era have higher expectations for environmental safety, service quality, and quality experiences of travel products, and they are more inclined to choose low-risk destinations with high trustworthiness and will also assess the safety risks associated with contact with others while traveling. Thus, travelers’ travel intentions are greatly influenced by their trust in the external environment.

Relying on social cognitive theory (SCT), this paper attempts to reveal the driving mechanism behind travel intention from the perspectives of institutional trust and interpersonal trust. Specifically, only on the basis of trust will tourists establish a good interaction with the destination (Chen and Phou, 2013), and thus generates travel intention. Therefore, tourists will show stronger travel intentions toward reliable destinations if they have trust in the safety of the destination. Furthermore, individuals’ trust in the external environment can serve as a subjective positive perception, which enhances their safety self-efficacy, and in turn increases their travel intentions. When faced with uncertainty in the external environment, different individuals will respond with different behaviors. Logically, psychological resilience (Block and Block, 2006) serves as a significantly differentiated personality trait that regulates the relationship between cognition and behavior (Block and Kremen, 1996), will alleviate tourists’ travel behaviors and concerns about health under travel consumption activities. In this way, psychological resilience might moderate the direct effect of trust on travel intention, and the indirect effect of trust through health risk perception.

## Proposed hypotheses and model

### Impact of trust on travel intention

Essentially, trust is a psychological state centered around a positive expectation that arises from the trustor’s willingness to take a certain risk to trust the trusted object (Mayer et al., 1995; Rousseau et al., 1998). According to Luhmann (2018), trust can be classified at the level of structural functionalism as interpersonal and institutional trust, where interpersonal trust refers to the degree of mutual emotion and awareness between individuals who are related by blood or have established some kind of relationship through interpersonal communication. The concept of institutional trust is based on the structure of social relations, incorporating legal regulations and political environments, which reduces the complexity of social interactions with external disciplinary and prevention mechanisms, including regulations, laws, and regulations. As a reflection of good government-public interaction, institutional trust is both a guarantee for the survival and development of the state polity, as well as its sociological and psychological basis. In a hierarchy of trust, individuals are governed first by their interpersonal relations with their families through socialization, secondly by their trusting of strangers they do not know, and finally by their trusting of political institutions, reflecting the spillover effect of interpersonal trust on institutional trust (Mishler and Rose, 2005; Wong et al., 2011). During the transition from traditional to modern societies, trust patterns shift, with interpersonal trust becoming institutional trust (Dowley and Silver, 2002).

As a subjective perception, trust arises from the trustor’s evaluation of the trustee’s past behavior. In order for trust to exist, certain conditions must be met, including mutual understanding between the parties and a willingness to take risks. Trust is a positive emotion in which the trustor has confidence in the trustee’s commitment, even if the trustee is faced with some uncertainty. Consumer trust is predicated on an environment of risk and uncertainty. Trust serves to reduce anxiety in customer decision dilemmas and to reduce transaction costs resulting from the search for and inspection of information (Kramer, 1999). As a perception of relative safety within a potentially hazardous environment, consumer trust can be viewed in two different ways: first, trust can be regarded as a subjective belief or expectation about the provider of a service or product (Lee and Back, 2008). Secondly, consumer trust implies the behavioral intention to rely on peers in a vulnerable situation. Widely present in everyday discourse, trust is one of the key resources for the development of modern societies (Freitag and Bühlmann, 2009) and plays a critical role in understanding interpersonal relationships, political systems, and travel development. The majority of travel takes place in unfamiliar environments and with unfamiliar relationships. When external rules cannot provide sufficient assurance that others will behave as we expect them to, trust can act as a subjective substitute for these rules, creating an environment conducive to open interpersonal interactions (Butler and Cantrell, 1994), an important variable in travel activities that may positively influence travelers’ risk assessments (Roth et al., 2016). A relationship of mutual trust is likely to develop when travelers connect themselves with other travelers (Hendrickson et al., 2011).

As per SCT, behavior is determined by individual cognition, individual behavior, and factors related to the environment in which it occurs, with these factors interacting dynamically and continuously to control human behavior through different operational mechanisms. Behaviors, environments, and individuals are all mutually determined, in which each acts as a mutual determinant. An important factor that hinders travel intention in the COVID-19 epidemic environment is the uncertainty of possible viral infection and physical isolation during travel. Social distance policies and wearing masks properly are significant in reducing this uncertainty, as well as effective epidemic prevention and control measures by destination authorities. It is only through trust that travelers will be able to develop a positive relationship with a destination (Chen and Phou, 2013), which in turn generates purchase intention (Sichtmann, 2007). Consequently, if travel destinations adopt the necessary measures for preventing and controlling epidemics and the public strictly abides by the relevant regulations, travelers will be more inclined to trust the destination. Through the interaction between individual cognition and environment, individuals show stronger convergence behavior toward reliable destinations, reducing the uncertainty surrounding their travel intentions. Accordingly, the hypothesis is formulated:

**H1:** Trust has a positive effect on travel intention.

**H1a:** Institutional trust has a positive effect on travel intention.

**H1b:** Interpersonal trust has a positive effect on travel intention.

### The mediating role of health risk perception

Risk leads to uncertainty and adverse consequences. When an individual’s behavior is completely predictable, there is no need for trust. In a risky environment, on the other hand, trust is crucial (Molm et al., 2000). Thus, when individuals perceive some level of risk, trust in the environment affects their behavior (Rousseau et al., 1998). Studies have shown that consumers’ perceptions of epidemics risk influence their attitudes and behaviors (Harris et al., 2018; Brewer and Sebby, 2021). Travel risk may encompass health, physical, psychological, financial, facility, social, and time, etc., (Huang et al., 2020). Among them, travel health risk perception is an assessment of the likelihood of travel-related health hazards occurring in a destination at a given time (Chien et al., 2017). In light of the COVID-19 epidemic, travel health risks are a focal concern for tourists, and health risks are closely linked to travel consumption decisions (Jonas et al., 2011). A study conducted by Rittichainuwat and Chakraborty (2009) revealed that travelers’ perceptions of health risks during the SARS outbreak negatively affected the travel industry in Thailand. Due to the effective prevention and control measures taken by the government in response to the COVID-19 epidemic, travelers developed appropriate institutional and interpersonal trust, which reduced uncertainty and health risk perceptions in travel activities, resulting in a higher travel intention. Therefore, the hypothesis is as followed:

**H2:** Health risk perception mediates the relationship between trust and travel intention.

### The mediating role of safety self-efficacy

Self-efficacy is the degree of confidence that an individual has in his or her own resilience and ability to cope, and is used to determine whether an individual has a complete risk coping mechanism. It can also be used as a tool to measure the confidence an individual has in organizing and executing actions to cope with stress in a risky situation (Maddux and Rogers, 1983). Social cognitive theory (Kanter, 2006) describes safety self-efficacy as an assessment of people’s ability to ensure their safety in complex environments and a cognitive construct that pushes them toward positive expectations about future events (Gavrilov-Jerković et al., 2014). Self-efficacy, as a belief, is strongly related to personal performance, and it influences individuals’ self-regulation of motivation and behavior through goal challenges and outcomes expectations (Bandura, 2002).

In research on the risk domain, researchers have suggested that people engage in certain activities by risk-averse behaviors and beliefs about safety and security (Binder et al., 2011). It has been shown that self-efficacy is closely related to individual behavior, and that the level of self-efficacy influences individuals’ strategies for achieving their goals and the decisions they make about how to attain them. Those who have high self-efficacy believe they can respond effectively to their environment and make appropriate choices, thus reducing the perception of potential negative outcomes (Rippetoe and Rogers, 1987; Zhou, 2011). In social public health events, the public’s safety self-efficacy contributes to their positive expectations of the social event, and it is dependent in part on the government’s response to the public health event (Lau et al., 2010). In the face of the COVID-19 epidemic, individuals’ trust in the external environment can serve as a subjective positive perception, an important source of enhancing safety self-efficacy, which in turn facilitates the tendency for these individuals to form positive travel consumption expectations. In light of this, the hypothesis was proposed:

**H3:** Safety self-efficacy has a mediating role between trust and travel intention.

### Moderating effect of psychological resilience

Psychological resilience is a protective psychological resource that enables individuals to adapt well facing with risk factors that may impede their development or pose a risk to their safety. It has been suggested (Block and Block, 2006) that psychological resilience is a static personality trait, while the more popular view is that it is influenced by both personality and environmental factors (Garcia-Dia et al., 2013). In the event of a setback or stressful situation, an individual may lose a certain amount of their resources. In addition to reducing the negative effects of stress, psychological resilience can assist individuals in gaining a better sense of control over their environment and assisting their recovery from difficult situations (Tugade and Fredrickson, 2004; Smith et al., 2008).

Psychological resilience is a significantly differentiated personality trait that regulates the relationship between cognition and behavior (Block and Kremen, 1996). Compared to individuals with low psychological resilience, individuals with high psychological resilience have richer internal resources and a greater sense of control over their environment, allowing them to better cope with dynamic environments (Shin et al., 2012). Travelers who possess psychological resilience as an intrinsic resource can help travelers enhance their judgment of their own adaptability, respond effectively to external environmental uncertainty, and have stronger endurance and higher self-regulatory ability to confront shocks and impacts caused by the COVID-19 epidemic. Travelers with high psychological resilience tend to be optimistic when assessing the environment and the risks of travel activities, alleviating concerns about certain negative consequences of travel consumption activities. In this way, trust may be moderated in its influence on travel intention. At the same time, the mediating effect of health risk perception on trust and travel intention may be moderated by psychological resilience. In this regard, the hypothesis was developed:

**H4:** Psychological resilience has a moderating effect on the relationship between trust and travel intention.

**H5:** Psychological resilience has a moderating effect on the health risk perception’s mediating role between trust and travel intention.

**FIGURE 1:**
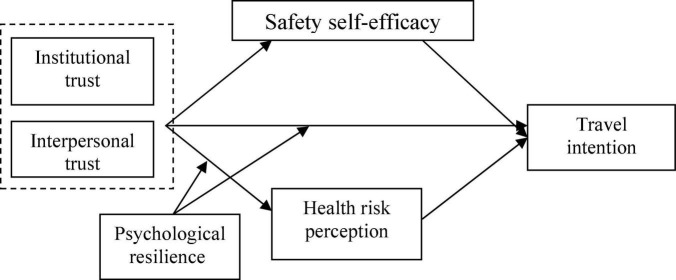
Research theoretical model.

The theoretical model is shown in Figure 1.

## Materials and methods

### Measures

The scales were all based on well-established scales in order to ensure their reliability and validity. The first part of the scale contains five subscales measuring trust, health risk perception, safety self-efficacy, psychological resilience, and travel intention. All items were rated on a 7-point Likert scale. In the second part, we provide demographic information.

#### Trust

We used four items adapted from Fancourt et al. (2020) and Nunkoo et al. (2012) to rate institutional trust. A sample item is “I believe the government is capable of preventing and controlling the epidemic.” We used four items adapted from Yamagishi and Yamagishi (1994) to rate interpersonal trust. A sample item is “I believe that most of my travel companions are trustworthy in their epidemic prevention measures.”

#### Health risk perception

We used five items adapted from Liu et al. (2016) and Chua et al. (2021). A sample item is “I am at risk of contracting COVID-19 while traveling.”

#### Safety self-efficacy

We used five items adapted from Bandura (1983), Fong et al. (2017), and Lau et al. (2011). A sample item is “Traveling during the epidemic, I am confident that I will not contract COVID-19.”

#### Psychological resilience

We used five items adapted from Lock et al. (2020). A sample item is “I believe I can recover from the stress of living with the epidemic.”

#### Travel intention

We used three items adapted from Zenker et al. (2019), Rastegar et al. (2021), and Wong and Yeh (2009). A sample item is “I am willing to travel to low-risk areas.”

### Sample and procedure

From June 2021 to September 2021, paper questionnaires were distributed on site or electronically in Chengdu and other Chinese cities. The research object in this paper is general consumers, so the questionnaire was administered without limiting the characteristics of participants. Following the principle of convenience sampling, we released on-site paper questionnaires for local participants in Chengdu, and we collected data online by using electronic questionnaires for participants in other regions. A total of 450 questionnaires were distributed and 409 were returned, with a return rate of 90.88%. A total of 379 valid questionnaires were obtained, with an effective rate of 84.22%, after excluding the questionnaires with more missing answers or regular answers (the total number of questions in the first part of the questionnaire was less than 20).

The gender distribution of the sample is relatively even, of which 54.9% of men and 45.1% of women were mainly 18–25 years old, accounting for 44.9%, followed by respondents aged 26–35 years old, accounting for 29.3%. In terms of education, 57.3% of respondents have a bachelor’s degree, while those with high school and below, specialist, master and above account for 14.8, 13.7, and 14.2%, respectively. As for monthly income, 4,001–6,000 yuan and 6,001–8,000 yuan were the main categories, accounting for 28.5 and 32.5%, respectively.

## Results

### External model testing

#### Model reliability evaluation

Cronbach’s alpha coefficient was used to measure the reliability of the scale. According to Table 1, all Cronbach’s alpha values exceeded 0.9, indicating high reliability. The composite reliability (CR) of the five latent variables were 0.916, 0.906, 0.924, 0.927, and 0.917, which were all greater than the baseline value of 0.70, which shows that the data between the items within the same dimension are reliable and consistent.

**TABLE 1:** Index system of PLS path analysis model.

Latent variables	Cronbach’s alpha	AVE	CR	Factor loading
Institutional trust	0.916	0.762	0.962	0.957
				0.860
				0.861
				0.864
Interpersonal trust				0.849
				0.844
				0.868
				0.874
Health risk perception	0.906	0.779	0.962	0.895
				0.887
				0.880
				0.868
Safety self-efficacy	0.924	0.858	0.948	0.865
				0.865
				0.870
				0.890
				0.888
Psychological resilience	0.927	0.766	0.943	0.901
				0.885
				0.880
				0.885
				0.845
Travel intention	0.917	0.773	0.945	0.925
				0.935
				0.919

#### Model validity evaluation

Based on the average variance extracted (AVE) values for the five latent variables, 0.762, 0.779, 0.858, 0.766, and 0.773 were all greater than the baseline value of 0.50, ensuring that the structure of the latent variables could explain at least 50% of the variance in the items, and the convergent validity of the measurement model was ideal. The square root of each of the five latent variables is greater than their absolute correlation coefficient, representing low correlation among the latent variables (shown in Tables 1, 2).

**TABLE 2:** Square root of average variance extracted (AVE) and correlation coefficients of latent variables.

	Trust	Safety self-efficacy	Travel intention	Safety self-efficacy	Psychological resilience
Trust	(0.873)				
Health risk perception	−0.865**	(0.883)			
Travel intention	0.865**	−0.812**	(0.926)		
Safety self-efficacy	0.864**	−0.847**	0.892**	(0.875)	
Psychological resilience	0.868**	−0.881**	0.923**	0.871**	(0.879)

Square Roots of AVE are reported in the parentheses on the diagonal.

**p < 0.01.

#### Multicollinearity and common method variance

Variance inflation factor (VIF) was examined to assess the degree of multicollinearity (O’brien, 2007). The VIF values for each predictor ranged from 1.000 to 4.631 (below 10), which indicates low levels of multicollinearity.

Further, we used Harman’s one-factor test to examine whether the issue of common method variance (CMV) is present (Podsakoff et al., 2003). By loading all of the variables into an exploratory factor analysis, we found that the first unrotated factor only accounted for 26.246% of the total variance in data. As it is well below the suggested cutoff (40%), there were no severe problem of CMV in this study.

### Internal model testing

#### Deterministic coefficient of the structural model (*R*^2^)

The primary indicator for PLS-SEM model assessment is the deterministic coefficient (*R*^2^), and an *R*^2^ of 0.75 or higher can be considered as a model with significant explanatory power. As shown in Table 3, the *R*^2^ of perceived health risk, safety self-efficacy, and travel intention were 0.851, 0.851, and 0.916, respectively, implying the strong explanatory power of the current model measurement variables for the latent variables.

**TABLE 3:** Deterministic coefficients of the structural model (*R*^2^).

	*R* ^2^	Adjusted *R*^2^
Health risk perception	0.853	0.851
Safety self-efficacy	0.810	0.809
Travel intention	0.917	0.916

#### Predictive relevance of structural models (*Q*^2^)

The predictive validity *Q*^2^ value is an important indicator to judge the predictive effectiveness of the model, and the closer the *Q*^2^ value is to 1, the higher the predictive relevance of the structural model. When the *Q*^2^ value is greater than 0.5, the model can be considered to have a fairly good predictive effect and a high degree of confidence. Using SmartPLS 3.0, the blindfolding calculation is conducted using the sample reuse technique. A portion of the data matrix is omitted during the calculation, and the omitted portion is estimated using the model. In total, 379 samples were used, and the default omitted distance seven cannot be divided by the total number of samples, which can be calculated directly. As can be seen from Table 4, the *Q*^2^ values of all conformations are greater than 0.5, signifying that the structural model has high predictive relevance.

**TABLE 4:** Predictive correlation of the structural model (*Q*^2^).

Trust	Health risk perception	Travel intention	Safety self-efficacy	Psychological resilience
0.684	0.615	0.669	0.640	0.650

### Hypothesis testing

#### Direct effects testing

We test our hypotheses by utilizing the Bootstrapping-based path analysis approach. Based on 5,000 bootstrap samples and 3,000 iterations, the bootstrap BCa method was used. We tested the path coefficients, *t*-statistics, and *p*-values between the independent variables and the dependent variable. The results are shown in Table 5.

**TABLE 5:** Bootstrapping results for test of direct effects.

	Original sample (O)	Sample mean (M)	Standard deviation (STDEV)	*t*-statistic (| O/STDEV|)	*P*-value
Trust →Travel intention	0.146	0.154	0.067	2.178	0.029
Institutional trust →Travel intention	0.556	0.555	0.055	10.074	0.000
Interpersonal trust →Travel intention	0.361	0.361	0.055	6.512	0.000
Trust →Health risk perception	–0.451	–0.454	0.078	5.791	0.000
Trust →Safety self-efficacy	0.383	0.388	0.069	5.520	0.000
Health risk perception →Travel intention	–0.222	–0.212	0.072	3.100	0.002
Safety self-efficacy →Travel intention	0.138	0.137	0.042	3.292	0.001

Bootstrap sample size = 5,000.

There is a significant positive effect of trust on travel intention, supporting hypothesis 1 (β = 0.146, *p* < 0.05). Both institutional trust and interpersonal trust are significantly positive factors influencing travel intention to different degrees, with institutional trust (β = 0.556, *p* < 0.001) showing a stronger effect than interpersonal trust (β = 0.361, *p* < 0.001). Based on these findings, hypotheses 1a and hypotheses 1b were supported. Table 5 shows that trust has a significant negative effect on health risk perception (β = −0.451, *p* < 0.001), health risk perception has a notable negative impact on travel intention (β = −0.222, *p* < 0.01), trust has a significant positive effect on safe self-efficacy (β = 0.383, *p* < 0.001), and safety self-efficacy has a salient positive effect on travel intention (β = 0.138, *p* < 0.001). As such, the paths from independent variables to mediation variables and from mediation variables to dependent variables are of heightened importance.

#### Mediation effects testing

Two mediating variables, health risk perception and safety self-efficacy, are added to the operation, using a bootstrapping method that does not require distribution assumptions, which has stable results regardless of whether it is applied to large or small samples (Preacher and Hayes, 2008).

Table 6 shows that the path “trust → health risk perception → travel intention” is significant at the 0.001 level, denoting the existence of a mediating effect, and hypothesis 2 was supported. The path “trust → safety self-efficacy → travel intention” is significant at the 0.01 level, and hypothesis 3 was supported.

**TABLE 6:** Bootstrapping results for test of mediation effects.

	Original sample (O)	Sample means (M)	Standard deviation (STDEV)	*t*-statistic (| O/STDEV|)	*P*-value
Trust →Health risk perception →Travel intention	0.100	0.094	0.030	3.401	0.001
Trust →Safety self-efficacy →Travel intention	0.053	0.053	0.019	2.836	0.005

Bootstrap sample size = 5,000.

#### Moderation effects testing

Using the Process plug-in developed by Hayes (2013) to determine the significance of the moderating variable of psychological resilience, the Bootstrapping algorithm found that the confidence intervals of the direct and conditional indirect effects did not contain 0, suggesting that the moderating and mediated effects of the moderated were significant. The calculated results are shown in Table 7, where the interaction term of trust and psychological resilience significantly and positively predicted travel intention (β = 0.147, *p* < 0.05), thus hypothesis 4 was supported.

**TABLE 7:** Bootstrapping results for test of moderation effect.

Effect type	Moderator	Level	Estimate	SE	95% confidence interval	
					Lower	Upper
Conditional direct effect	Psychological Resilience	M−1SD	0.172	0.036	0.101	0.243
		M + 1SD	0.121	0.043	0.038	0.205
Conditional indirect effects		M−1SD	–0.498	0.041	–0.579	–0.417
		M + 1SD	–0.349	0.056	–0.458	–0.239

Bootstrap sample size = 5,000.

To further explore the moderating role of psychological resilience between trust and travel intention, a simple slope test was used to obtain high (+1 SD) and low (−1 SD) levels of psychological resilience. The results show (see Table 7) that trust has a significant positive predictive effect on travel intention for subjects with low psychological resilience (estimate = 0.172, 95% CI [0.101, 0.243], excluding zero) and the same positive predictive effect of trust on travel intention for subjects with high psychological resilience (estimate = 0.121, 95% CI [0.038, 0.205], excluding zero), with this effect diminishing as the level of psychological resilience increases.

**FIGURE 2:**
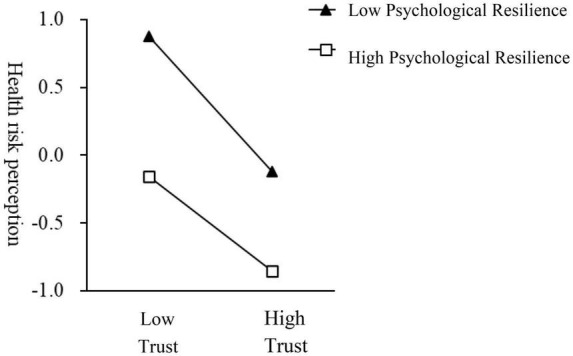
Moderating effect of psychological resilience on the relationship between trust and travel intention.

Table 7 shows the results of the conditional indirect effects test, and Figure 2 shows the interaction results. The indirect effect of trust on travel intention via health risk perceptions was significant at different levels of psychological resilience, with a significant negative predictive effect of trust on health risk perceptions for the low group of tourists (estimate = −0.498, 95% CI [−0.579, −0.417], excluding zero), and a significant negative predictive effect of trust on travel intention for the high group of tourists, with a relatively small predictive effect (estimate = −0.349, 95% CI [−0.458, −0.239], excluding zero). Hypothesis 5 was proved.

## Discussion and implications

### Theoretical implications

A significant and positive correlation is observed between travelers’ trust and their travel intention, which supports the views of Ekinci and Hosany (2006). Based on Luhmann’s study in combination with China’s prevention and control practices for the COVID-19 epidemic, this paper further subdivides trust into institutional trust and interpersonal trust, i.e., trust that the government and the public can continuously prevent and control the epidemic to ensure the safety of the tourism environment. This empirical study finds that institutional trust has a greater influence on travel intention than interpersonal trust, suggesting that travelers’ trust in the government’s measures and the ability of the government to prevent and control epidemics is a crucial factor in travel intention.

According to the theory of social information processing, individual activities and behaviors do not occur in a vacuum, and are usually affected by complex and fuzzy social situations (Salancik and Pfeffer, 1978). The social environment in which individuals live, provides a variety of information that affects his attitude and behavior. By processing and interpreting specific social information, individuals decide what kind of attitude and behavior to adopt (Lau and Liden, 2008). Without a doubt that under the COVID-19, the social environment faced by individuals is uncertain and complex, and they rely more on the effective information provided by social environment. As a public authority, the government plays an important role in dealing with social public affairs and maintaining public health security. Therefore, the epidemic prevention announcement, epidemic prevention system and measures taken by the government not only directly affect the prevention and control effect of COVID-19, but also are incorporated into individual information processing systems as important information sources. The perception of institutional trust affects the attitude and behavior of individuals such as travel intention.

This supports institutionalist scholars’ assertion that “trust depends on macro policy formulation, and citizens’ evaluation of the effectiveness of these policies’ implementation.” Traveler trust can indirectly drive travel intention through health risk perception and safety self-efficacy. In the COVID-19 epidemic environment where risk and uncertainty coexist, trust in the external environment can effectively reduce travelers’ health risk perceptions and enhance safety self-efficacy. A dynamic decision relationship exists between the environment, perceptions, and behavioral intentions of travelers, and when travelers’ evaluations of the environment and self-perceptions are positive, that can have a vital impact on their travel intention, which is consistent with the basic assumptions of social cognitive theory on individual behavioral decision making.

Psychological resilience moderates the strength of the relationship between travelers’ trust and travel intention. Trust has a greater positive effect on travel intention among tourists with low psychological resilience than among those with high psychological resilience. In addition, psychological resilience may moderate the impact of travelers’ perception of health risks on their travel intention. During the COVID-19 epidemic, psychological resilience can assist individuals in gaining internal resources that allow them to adapt proactively to external stresses. This can be done by adjusting perceptions about the environment, such as trust, in an effort to reduce resource imbalances due to uncertainty and risk perceptions.

### Practical implications

In the first place, governance of major public health events should take full advantage of the strengths of a responsive government. Adopting effective preventive and control measures for the COVID-19 epidemic, especially improving laws, regulations, and special policies for managing major public health events in response to the unconventional nature of the COVID-19 epidemic, can not only guarantee public health physically, but also gives the public the confidence to overcome the epidemic spiritually. Societies can only be safer and more trustworthy if a scientific epidemic prevention system is in place and norms are implemented, bringing predictability to individual travel consumption behavior.

Additionally, it’s important to maintain a safe travel environment and ensure the security of travel intention. Health risk perception, safety self-efficacy, etc., are essentially travelers’ demands for safe travel, and therefore, travel intention recovery necessitates a sense of psychological safety for travelers. It is imperative that travel destinations take visible measures to construct a realistic defense against the epidemic. At the same time, flexible communication strategies should be adopted to provide travelers with the psychological resources to cope with the uncertainty brought about by the epidemic. Travel attractions, for instance, should strengthen their daily management to ensure that personnel protection measures are in place, strictly implement a reservation system for tickets, comply with regulations related to the number of travelers received at the attractions, control the scale of reception, and avoid the gathering of people. Through what they observe and hear, travelers can gain a sense of trust and security in the travel environment mentioned above.

### Limitations and future research directions

Despite the fact that trust is analyzed as a single dependent variable in this paper, the process of forming and achieving a travel intention is very complex in realistic scenarios, which is caused by a combination of environmental cognitive factors, including trust, positive thoughts, stress perception, and emotional overflow. Trust, as an antecedent variable, is not yet a complete explanation of the dynamics of the formation of travel intention. In addition, Chinese culture tends toward collectivism and power distance, which will result in a different trust in institutions and interpersonal trust from western cultures, which should be explored from a cross-cultural perspective in depth. In this paper, only one study was conducted; however, the mechanism of trust’s influence on travel intention displays dynamic changes due to the external and internal environment, and the results may differ in different contexts. Multi-point stratification can be adopted to test the theoretical model of trust-travel intention in the future. Furthermore, the sample size of the study is relatively small. Data collection during the COVID-19 epidemic was limited by geographical and spatial constraints, and the sample was collected primarily using web-based questionnaires, which had limitations in terms of size and applicability. Future studies could test the applicability of the research model by selecting regions with different COVID-19 epidemic risk levels to conduct large sample comparison studies.

## Data availability statement

The original contributions presented in this study are included in the article/supplementary material, further inquiries can be directed to the corresponding author/s.

## Ethics statement

The studies involving human participants were reviewed and approved by the Southwestern University of Finance and Economics, although the school did not have a formal institutional review board. Written informed consent for participation was not required for this study in accordance with the national legislation and the institutional requirements.

## Author contributions

LW performed conceptualization, methodology, writing – original draft, review and editing, and project administration. WL performed conceptualization, methodology, writing – original draft, and review and editing. GC and YZ performed methodology, writing – original draft, and review and editing. JW and YL performed supervision and writing – review and editing. All authors contributed to the article and approved the submitted version.

## References

[B1] Bandura A. (1983). Self-efficacy determinants of anticipated fears and calamities. *J. Pers. Soc. Psychol.* 2 464–469.

[B2] Bandura A. (2002). Social cognitive theory in cultural context. *Appl. Psychol.* 2 269–290. 10.1111/1464-0597.00092

[B3] Binder A. R. Scheufele D. A. Brossard D. Gunther A. C. (2011). Interpersonal amplification of risk? citizen discussions and their impact on perceptions of risks and benefits of a biological research facility. *Risk Anal.* 2 324–334. 10.1111/j.1539-6924.2010.01516.x 21039705

[B4] Block J. Block J. H. (2006). Venturing a 30-year longitudinal study. *Am. Psychol.* 4 315–327. 10.1037/0003-066X.61.4.315 16719676

[B5] Block J. Kremen A. M. (1996). IQ and ego-resiliency: Conceptual and empirical connections and separateness. *J. Pers. Soc. Psychol.* 2 349–361. 10.1037/0022-3514.70.2.349 8636887

[B6] Bradbury-Jones C. Isham L. (2020). The pandemic paradox: The consequences of COVID-19 on domestic violence. *J. Clin. Nurs.* 1 2047–2049. 10.1111/jocn.15296 32281158 PMC7262164

[B7] Brewer P. Sebby A. G. (2021). The effect of online restaurant menus on consumers’ purchase intentions during the COVID-19 pandemic. *Int. J. Hosp. Manag.* 94:102777. 10.1016/j.ijhm.2020.102777 34785837 PMC8588438

[B8] Butler J. K. Jr. Cantrell R. S. (1994). Communication factors and trust: An exploratory study. *Psychol. Rep.* 1 33–34. 10.2466/pr0.1994.74.1.33

[B9] Chen C. F. Phou S. (2013). A closer look at destination: Image, personality, relationship and loyalty. *Tour. Manag.* 36 269–278. 10.1016/j.tourman.2012.11.015

[B10] Chien P. M. Sharifpour M. Ritchie B. W. Watson B. (2017). Travelers’ health risk perceptions and protective behavior: A psychological approach. *J. Travel Res.* 6 744–759. 10.1177/0047287516665479

[B11] Chua B. L. Al-Ansi A. Lee M. J. Han H. (2021). Impact of health risk perception on avoidance of international travel in the wake of a pandemic. *Curr. Issues Tour.* 7 985–1002. 10.1080/13683500.2020.1829570

[B12] Dowley K. M. Silver B. D. (2002). Social Capital, Ethnicity and Support for Democracy in the Post-Communist States. *Eur. Asia Stud.* 4 505–527. 10.1080/09668130220139145

[B13] Ekinci Y. Hosany S. (2006). Destination personality: An application of brand personality to tourism destinations. *J. Travel Res.* 2 127–139. 10.1177/0047287506291603

[B14] Fancourt D. Steptoe A. Wright L. (2020). The Cummings effect: Politics, trust, and behaviours during the COVID-19 pandemic. *Lancet* 10249 464–465. 10.1016/S0140-6736(20)31690-1 PMC7613216 32771083

[B15] Fong S. F. Lo M. C. Songan P. Nair V. (2017). Self-efficacy and sustainable rural tourism development: Local communities’ perspectives from Kuching, Sarawak. *Asia Pac. J. Tour. Res.* 2 147–159. 10.1080/10941665.2016.1208668

[B16] Freitag M. Bühlmann M. (2009). Crafting trust: The role of political institutions in a comparative perspective. *Comp. Political Stud.* 12 1537–1566. 10.1177/0010414009332151

[B17] Garcia-Dia M. J. DiNapoli J. M. Garcia-Ona L. Jakubowski R. O’Flaherty D. (2013). Concept analysis: Resilience. *Arch. Psychiatr. Nurs.* 6 264–270. 10.1016/j.apnu.2013.07.003 24238005

[B18] Gavrilov-Jerković V. Jovanović V. Žuljević D. Brdarić D. (2014). When less is more: A short version of the personal optimism scale and the self-efficacy optimism scale. *J. Happiness Stud.* 2 455–474. 10.1007/s10902-013-9432-0

[B19] Harris K. J. Ali F. Ryu K. (2018). Foodborne illness outbreaks in restaurants and patrons’ propensity to return. *Int. J. Contemp. Hosp.* 3 1273–1292. 10.1108/IJCHM-12-2016-0672

[B20] Hayes A. F. (2013). *Introduction to mediation, moderation, and conditional process analysis: A regression-based approach.* New York, NY: Guilford Press.

[B21] Hendrickson B. Rosen D. Aune R. K. (2011). An analysis of friendship networks, social connectedness, homesickness, and satisfaction levels of international students. *Int. J. Intercult. Relat.* 3 281–295. 10.1016/j.ijintrel.2010.08.001

[B22] Huang X. Dai S. Xu H. (2020). Predicting tourists’ health risk preventative behaviour and travelling satisfaction in Tibet: Combining the theory of planned behaviour and health belief model. *Tour. Manag. Perspect.* 33:100589. 10.1016/j.tmp.2019.100589

[B23] Jonas A. Mansfeld Y. Paz S. Potasman I. (2011). Determinants of health risk perception among low-risk-taking tourists traveling to developing countries. *J. Travel Res.* 1 87–99. 10.1177/0047287509355323

[B24] Kanter R. M. (2006). *Confidence: How Winning Streaks and Losing Streaks Begin and End.* New York, NY: Random House.

[B25] Kim S. W. Su K. P. (2020). Using psychoneuroimmunity against COVID-19. *Brain Behav. Immun.* 87 4–5. 10.1016/j.bbi.2020.03.025 32234338 PMC7194899

[B26] Kramer R. M. (1999). Trust and distrust in organizations: Emerging perspectives, enduring questions. *Annu. Rev. Psychol.* 50:569. 10.1146/annurev.psych.50.1.569 15012464

[B27] Lau D. C. Liden R. C. (2008). Antecedents of coworker trust: Leaders’ blessings. *J. Appl. Psychol*. 93 1130–1138. 10.1037/0021-9010.93.5.1130 18808230

[B28] Lau J. T. F. Griffiths S. Au D. W. H. Choi K. C. (2011). Changes in knowledge, perceptions, preventive behaviours and psychological responses in the pre-community outbreak phase of the H1N1 epidemic. *Epidemiol. Infect.* 1 80–90. 10.1017/S0950268810001925 20800008

[B29] Lau J. T. F. Griffiths S. Choi K. C. Tsui H. Y. (2010). Avoidance behaviors and negative psychological responses in the general population in the initial stage of the H1N1 pandemic in Hong Kong. *BMC Infect. Dis.* 1:139. 10.1186/1471-2334-10-139 20509887 PMC2891756

[B30] Lee J. S. Back K. J. (2008). Attendee-based brand equity. *Tour. Manag.* 2 331–344. 10.1016/j.tourman.2007.03.002

[B31] Liu B. Pennington-Gray L. Krieger J. (2016). Tourism crisis management: Can the extended parallel process model be used to understand crisis responses in the cruise industry? *Tour. Manag.* 55 310–321. 10.1016/j.tourman.2016.02.021

[B32] Lock S. Rees C. S. Heritage B. (2020). Development and validation of a brief measure of psychological resilience: The state–trait assessment of resilience scale. *Aust. Psychol.* 1 10–25. 10.1111/ap.12434

[B33] Luhmann N. (2018). *Trust and power.* Hoboken, NJ: John Wiley and Sons.

[B34] Maddux J. E. Rogers R. W. (1983). Protection motivation and self-efficacy: A revised theory of fear appeals and attitude change. *J. Exp. Soc. Psychol.* 5 469–479. 10.1016/0022-1031(83)90023-9

[B35] Mayer R. C. Davis J. H. Schoorman F. D. (1995). An integrative model of organizational trust. *Acad. Manag. Rev.* 3 709–734. 10.5465/amr.1995.9508080335

[B36] Mishler W. Rose R. (2005). What are the political consequences of trust? A test of cultural and institutional theories in Russia. *Comp. Polit. Stud.* 9 1050–1078. 10.1177/0010414005278419

[B37] Molm L. D. Takahashi N. Peterson G. (2000). Risk and trust in social exchange: An experimental test of a classical proposition. *Am. J. Sociol.* 5 1396–1427. 10.1086/210434

[B38] Nakayachi K. Cvetkovich G. (2010). Public trust in government concerning tobacco control in Japan. *Risk Anal.* 1 143–152. 10.1111/j.1539-6924.2009.01306.x 19878484

[B39] Nunkoo R. Ramkissoon H. Gursoy D. (2012). Public trust in tourism institutions. *Ann. Tour. Res.* 3 1538–1564. 10.1016/j.annals.2012.04.004

[B40] O’brien R. M. (2007). A caution regarding rules of thumb for variance inflation factors. *Qual. Quant.* 41 673–690. 10.1007/s11135-006-9018-6

[B41] Podsakoff P. M. Mackenzie S. B. Lee J. Y. Podsakoff N. P. (2003). Common method biases in behavioral research: A critical review of the literature and recommended remedies. *J. Appl. Psychol.* 88 879–903. 10.1037/0021-9010.88.5.879 14516251

[B42] Preacher K. J. Hayes A. F. (2008). Asymptotic and resampling strategies for assessing and comparing indirect effects in multiple mediator models. *Behav. Res. Methods* 3 879–891. 10.3758/BRM.40.3.879 18697684

[B43] Rastegar R. Seyfi S. Rasoolimanesh S. M. (2021). How COVID-19 case fatality rates have shaped perceptions and travel intention? *J. Hosp. Tour. Manag.* 47 353–364. 10.1016/j.jhtm.2021.04.006

[B44] Rippetoe P. A. Rogers R. W. (1987). Effects of components of protection-motivation theory on adaptive and maladaptive coping with a health threat. *J. Pers. Soc. Psychol.* 3 596–604. 10.1037/0022-3514.52.3.596 3572727

[B45] Rittichainuwat B. N. Chakraborty G. (2009). Perceived travel risks regarding terrorism and disease: The case of Thailand. *Tour. Manag.* 3 410–418. 10.1016/j.tourman.2008.08.001

[B46] Roth B. Trautmann S. T. Voskort A. (2016). The role of personal interaction in the assessment of risk attitudes. *J. Behav. Exp. Econ.* 63 106–113. 10.1016/j.socec.2016.06.004

[B47] Rousseau D. M. Sitkin S. B. Burt R. S. Camerer C. (1998). Not so different after all: A cross-discipline view of trust. *Acad. Manag. Rev.* 3 393–404. 10.5465/amr.1998.926617

[B48] Salancik G. R. Pfeffer J. (1978). A social information processing approach to job attitudes and task design. *Adm. Sci. Q.* 23 224–253. 10307892

[B49] Shin J. Taylor M. S. Seo M. G. (2012). Resources for change: The relationships of organizational inducements and psychological resilience to employees’ attitudes and behaviors toward organizational change. *Acad. Manag. J.* 3 727–748. 10.5465/amj.2010.0325

[B50] Sichtmann C. (2007). An analysis of antecedents and consequences of trust in a corporate brand. *Eur. J. Mark.* 41 999–1015. 10.1108/03090560710773318

[B51] Smith B. W. Dalen J. Wiggins K. Tooley E. Christopher P. Bernard J. (2008). The brief resilience scale: Assessing the ability to bounce back. *Int. J. Behav. Med.* 3 194–200. 10.1080/10705500802222972 18696313

[B52] Tugade M. M. Fredrickson B. L. (2004). Resilient individuals use positive emotions to bounce back from negative emotional experiences. *J. Pers. Soc. Psychol.* 2 320–333. 10.1037/0022-3514.86.2.320 14769087 PMC3132556

[B53] Wong J. Y. Yeh C. (2009). Tourist hesitation in destination decision making. *Ann. Tour. Res.* 1 6–23. 10.1016/j.annals.2008.09.005

[B54] Wong T. K. Wan P. Hsiao H. M. (2011). The bases of political trust in six Asian societies: Institutional and cultural explanations compared. *Int. Political Sci. Rev.* 3 263–281. 10.1177/0192512110378657

[B55] Yamagishi T. Yamagishi M. (1994). Trust and commitment in the United States and Japan. *Motiv. Emot.* 2 129–166. 10.1007/BF02249397

[B56] Zenker S. von Wallpach S. Braun E. Vallaster C. (2019). How the refugee crisis impacts the decision structure of tourists: A cross-country scenario study. *Tour. Manag.* 71 197–212. 10.1016/j.tourman.2018.10.015

[B57] Zhou T. (2011). Understanding online community user participation: A social influence perspective. *Internet Res.* 1 67–81. 10.1108/10662241111104884

